# Easy and Rapid Binding Assay for Functional Analysis of Disulfide-Containing Peptides by a Pull-Down Method Using a Puromycin-Linker and a Cell-Free Translation System

**DOI:** 10.3390/biology4010161

**Published:** 2015-03-02

**Authors:** Yutaro Tanemura, Yuki Mochizuki, Shigefumi Kumachi, Naoto Nemoto

**Affiliations:** Graduate School of Science and Engineering, Saitama University, Sakura-ku, Saitama 338-8570, Japan; E-Mails: s14mp224@mail.saitama-u.ac.jp (Y.T.); mochiduki.yuki@gmail.com (Y.M.); kumachi0605@gmail.com (S.K.)

**Keywords:** molecular interaction analysis, pull-down assay, cell-free translation system, puromycin, constrained peptide, cyclic peptide, disulfide-rich peptide, cross-linking, *in vitro* selection, directed evolution

## Abstract

Constrained peptides are an attractive class as affinity reagents or drug leads owing to their excellent binding properties. Many kinds of these peptides, such as cyclic peptides containing disulfide bridges, are found in nature or designed artificially by directed evolution. However, confirming the binding properties of the disulfide-rich peptides can be generally difficult, because of oxidative folding problems in the preparation steps. Therefore, a method for evaluating the binding properties of such peptides rapidly and easily is required. Here, we report an easy and rapid method for preparing biotin-attached peptides containing disulfide bridges or a chemical cross-linker using a cell-free translation system and a puromycin-linker, which is applicable to pull-down assays for protein (or peptide) molecular interaction analysis.

## 1. Introduction

Cyclic peptides including disulfide-rich peptides have emerged as an important class of drug leads for the development of peptide-based therapeutics [1,2]. Disulfide-rich peptides are one of the primary categories of cyclic peptides in nature, which are found in a variety of fungi, plants and animals [3,4]. Natural disulfide-rich peptides frequently exhibit a wide variety of potent biological activities, such as channel blocking, enzyme inhibition, antimicrobial and anticancer activities [3,4]. They have a particular and well-defined folded structure, stabilized mainly by the formation of two or more disulfide bonds. The loop regions have been shown to adapt diverse amino acid sequences, which enable them to bind to a variety of target proteins by altering the loop region sequences [3]. Furthermore the constrained structures frequently have exceptional proteolytic, chemical and thermal stability [5]. These features make them promising molecular scaffolds for drug leads and diagnostic reagents [1,6].

Several kinds of disulfide-rich peptides have been explored by high throughput proteomic and transcriptomic approaches, or a combination of these methods from natural recourses, such as venom of scorpions, spiders, or cone snails [7,8]. Beyond that, improvement of native disulfide-rich peptides’ properties has been studied. For example, the matriptase inhibitory activity and selectivity of sunflower trypsin inhibitor-1 and Momordica cochinchinensis trypsin inhibitor-II were enhanced by adding point mutations based on structure-activity relationship analyzed by alanine scanning [9]. Furthermore, known disulfide-rich peptides have been used as scaffolds to mimic the function of a protein by grafting an epitope [10], enhance the activity of bioactive peptides [11], improve the inserted peptide stability in human serum [12], and have been engineered by directed evolution methods to have molecular recognition properties [13,14]. Additionally, *de novo* designs of disulfide-rich peptide binding to target proteins were examined by *in vitro* display technologies, such as cDNA display and mRNA display [15,16,17,18]. The binding properties of many kinds of disulfide-rich peptides have been studied as described above. However, the molecular interaction of disulfide-rich peptides is difficult to study, because of their oxidative folding problems [19]. For example, peptides with more than two disulfide bonds can have several disulfide patterns, which cannot be determined from the amino acid sequence. Even when the correct disulfide binding pattern is clear, production of disulfide-rich peptides is complicated, although they can be produced by *Escherichia coli* or chemical synthesis [20].

Previously, we have developed a pull-down method using biotin-attached peptides prepared with a cell-free translation system and a puromycin-linker [21]. In the pull-down method small quantities of a biotin-attached peptide, which are enough to confirm their affinity against target proteins, are synthesized from its coding mRNA-linker fusion molecule using the cell-free translation system. The pull-down method is a good choice for analyzing the binding properties of many candidate peptides and variants rapidly, easily and at low cost. In this study, we report that this pull-down method can be applied to easily and rapidly analyze the interaction between the disulfide-rich peptides and the target molecules.

Although disulfide-containing peptides are a highly attractive class of cyclic peptides, they can only be used under oxidative conditions, which restrict their applications [22]. To circumvent this issue, substitution of the disulfide bridges with other linking forms has been studied [22,23]. It is important that the pull-down method is applicable for evaluating the binding properties of cyclic peptides containing non-disulfide cross-linking. In this study, our abovementioned pull-down method was applied to evaluate the binding properties of a peptide containing disulfide bridges or a chemical cross-linker.

## 2. Experimental Section

### 2.1. Pull-Down Method for Disulfide-Containing Peptides

**Figure 1:**
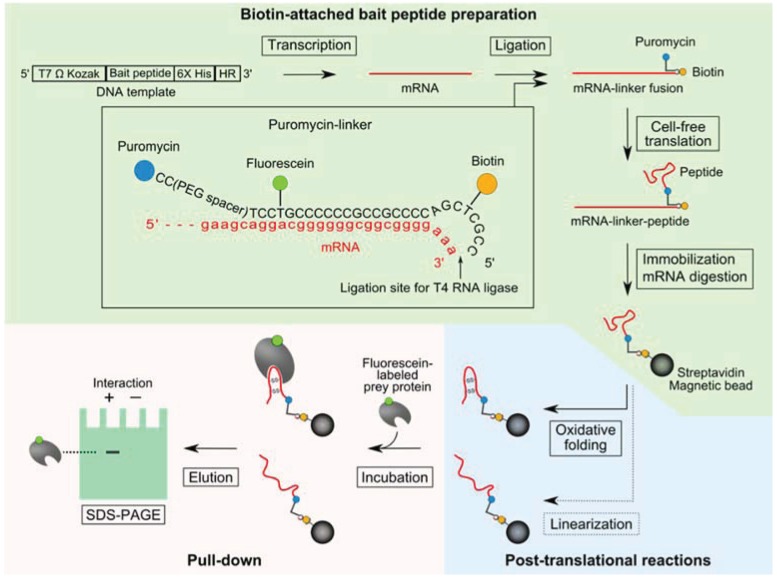
Schematic of a pull-down method using a puromycin-linker with a cell-free translation system for binding analysis of disulfide-containing peptides. The steps for biotin-attached peptide preparation, post-translational reactions, and pull-down are highlighted by light green, light blue and light red respectively. Bait peptide-coding DNA template is transcribed into mRNA by T7 polymerase. A puromycin-linker is hybridized to the mRNA,and the 5'-terminus of the puromycin-linker and 3'-terminus of the mRNA are ligated with T4 RNA ligase and T4 PNK. The ligation product is translated by the cell-free translation system, and then a fusion reaction of puromycin with the nascent peptide is facilitated by adding KCl and MgCl_2_. The translation product is immobilized on streptavidin magnetic beads and the mRNA portion of the translation product is digested with RNase H. Oxidative folding of each immobilized bait peptide is performed in the presence of reduced glutathione (GSH), oxidized glutathione (GSSG) and protein disulfide isomerase (PDI). Iodoacetamide-reacted bait peptide is prepared as a negative control. Fluorescein-labeled prey protein solution is incubated with each SA-bead-immobilized bait peptide and bound prey proteins are resolved by 4% stacking-15% separating SDS-PAGE and visualized by a fluorimager.

A schematic of the pull-down method and the puromycin-linker construct is shown in Figure 1. The synthesized puromycin-linker was purchased from Tsukuba Oligo Service (Tsukuba, Japan). The bait peptide-coding DNA template comprised of a T7 promoter, Omega sequence, Kozak sequence, bait-peptide coding region, hexa-histidine-tag, and hybridizing region (HR) of the puromycin-linker (Figure 1). Sequences of disulfide-containing peptide aptamers against soluble interleukin-6 receptor (sIL-6R): Cys2-6 and Cys4-2 were obtained from a previous report [17].

Biotin-attached peptide was prepared as follows: DNA was transcribed to mRNA using the T7 RiboMAX Express Large Scale RNA Production System (Promega, Madison, WI, USA), and the synthesized mRNA was purified with an After Tri Reagent RNA Clean-up Kit (Favorgen, Ping-Tung, Taiwan). The puromycin-linker was hybridized to the purified mRNA, and the 5'-terminus of the puromycin-linker and the 3'-terminus of the mRNA were ligated with T4 RNA ligase (Takara Bio, Otsu, Japan) and polynucleotide kinase (PNK; Takara Bio) at 25 °C for 1 h. Six picomoles of the ligation product were placed in 50 µL of a cell-free translation reaction solution with the Retic Lysate IVT kit (Thermo Fisher Scientific, Waltham, MA, USA) and incubated at 30 °C for 30 min. Then, 20 µL of 3 M KCl and 6 µL of 1 M MgCl_2_ were added to the reaction solution and incubated at 37 °C for 1 h. Eighteen microliters of EDTA solution (0.5 M, pH 8.0) were added to the translation reaction and incubated at 25 °C for 10 min to remove bound ribosomes. The mRNA-linker-peptide fusion molecules were isolated from the translation reaction solution using 30 μL of Dynabeads MyOne Streptavidin C1 (SA-beads; Thermo Fisher Scientific) according to the supplier’s instructions. RNase H (Takara Bio) was added to the sample and incubated at 37 °C for 30 min, to degrade the mRNA portion of the fusion molecule.

Post-translational reactions were performed as follows: The immobilized peptides were reduced with phosphate-buffered saline (PBS) containing 10 mM of Tris(2-carboxyethyl)phosphine (TCEP; Thermo Fisher Scientific) at 25 °C for 5 min. Then, the buffer was replaced with a folding buffer [50 mM Tris-HCl, pH 7.6, 100 mM NaCl, 1 mM EDTA, 10 mM reduced glutathione (GSH), 1 mM oxidized glutathione (GSSG), 0.1% Tween-20, and protein disulfide isomerase (PDI) at an equimolar ratio with the input mRNA] and incubated at 25 °C for 1 h for oxidative folding. Additionally, 2-iodoacetamide (final conc. 10 mM) was added to the TCEP-treated peptide samples to prepare linear peptides.

Pull-down of prey protein and detection were performed as follows: Recombinant human sIL-6R was purchased from ACRO Biosystems (Beijing, China) and used as the prey protein. The prey protein was labeled with N-hydroxysuccinimide fluorescein (Thermo Fisher Scientific) at a ratio of >1.0 dye/protein. The resulting fluorescein-labeled sIL-6R solution (200 nM) was incubated with the SA-bead-immobilized bait peptide at 25 °C for 1 h in PBS containing 0.1% Tween-20 (PBS-T). After three washes with PBS-T, the residual prey proteins were eluted by addition of sodium dodecyl sulfate polyacrylamide gel electrophoresis (SDS-PAGE) sample buffer and incubated at 90 °C for 3 min. The eluates were resolved by 4% stacking-15% separating SDS-PAGE and visualized by a fluorimager (PharosFX; Bio-Rad, Hercules, CA, USA). The band intensity in each lane was measured using Quantity One 1-D Analysis Software (Bio-Rad). The band intensities were normalized against background of the polyacrylamide gel and calculated as the total band intensity of the each lane was 100%.

### 2.2. Introduction of a Chemical Cross-Linker into a Disulfide-Containing Peptide

**Figure 2:**
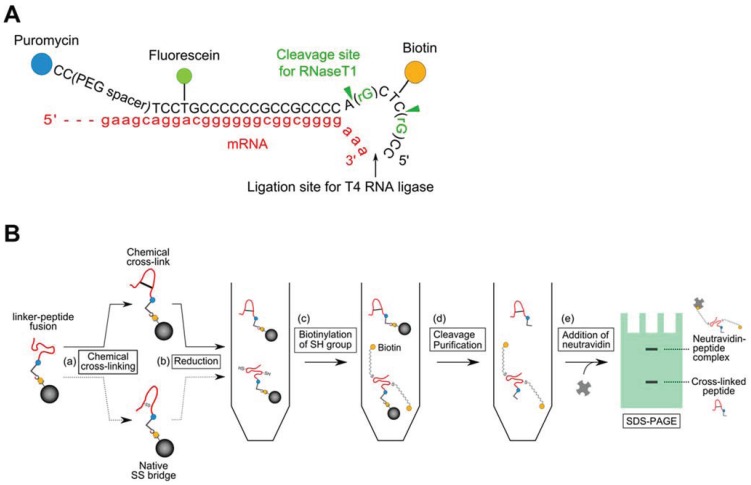
Introduction of a chemical cross-linker into a disulfide-containing peptide. (**A**) A modified puromycin-linker construct for estimation of the chemical cross-linking efficiency by a gel shift assay; (**B**) Schematic of the gel-shift assay. (**a**) The Cys2-6 peptides on the SA-beads were reacted with bis(maleimido)ethane (BMOE) in the presence of Tris(2-carboxyethyl)phosphine (TCEP). (**b**) The BMOE-treated peptides were reduced again with TCEP. (**c**) The TCEP-treated peptides were reacted with maleimide-PEG_11_-Biotin reagent to biotinylate free SH groups. (**d**) The peptide-linker fusion molecules were released from the SA-beads by RNase T1 treatment, followed by purification using the hexa-histidine-tag in the peptide. (**e**) Neutravidin was added to the sample and the mixture was resolved by 4% stacking-15% separating SDS-PAGE and visualized by a fluorimager.

A schematic of a gel-shift assay for estimation of the chemical cross-linking efficiency is shown in Figure 2. SA-bead-immobilized Cys2-6 peptides were prepared in the same manner as the abovementioned procedure, but with the modified puromycin-linker which contains two guanine ribonucleotides shown in Figure 2A. The Cys2-6 peptides were treated with 10 mM TCEP in a conjugation buffer [100 mM phosphate, pH 7.2, 150 mM NaCl, 10 mM EDTA, 0.025% Tween-20] at 25 °C for 5 min, to reduce the disulfide bridge. Then, bis(maleimido)ethane (BMOE; Thermo Fisher Scientific) solution was added to the sample at the indicated concentration and incubated at 25 °C for 1 h. After washing the SA-beads twice, the non-reacted Cys2-6 peptides were reduced with TCEP again, and then maleimide-PEG_11_-Biotin (Thermo Fisher Scientific) solution (final conc. 10 mM) was added to the sample and incubated at 25 °C for 2.5 h. The Cys2-6 peptide-linker fusion molecules were released from the beads by RNase T1 (Thermo Fisher Scientific) at 37 °C for 15 min. Each supernatant containing Cys2-6 peptide-linker fusion molecules was incubated with 10 μL of His Mag Sepharose Ni magnetic beads (GE Healthcare, Pittsburgh, PA, USA) at 25 °C for 1 h. The Ni-NTA magnetic beads were washed three times and the remaining peptides were eluted according to the supplier’s instructions. Neutravidin solution (final conc. 20 μM; Thermo Fisher Scientific) was added to each eluate and incubated at 25 °C for 30 min. These samples were resolved by 4% stacking-15% separating SDS-PAGE and visualized by a fluorimager. The band intensity was measured using the Quantity One 1-D Analysis Software.

## 3. Results and Discussion

### 3.1. Evaluation of the Interaction between the Disulfide-Containing Peptides and the Target Protein

**Figure 3:**
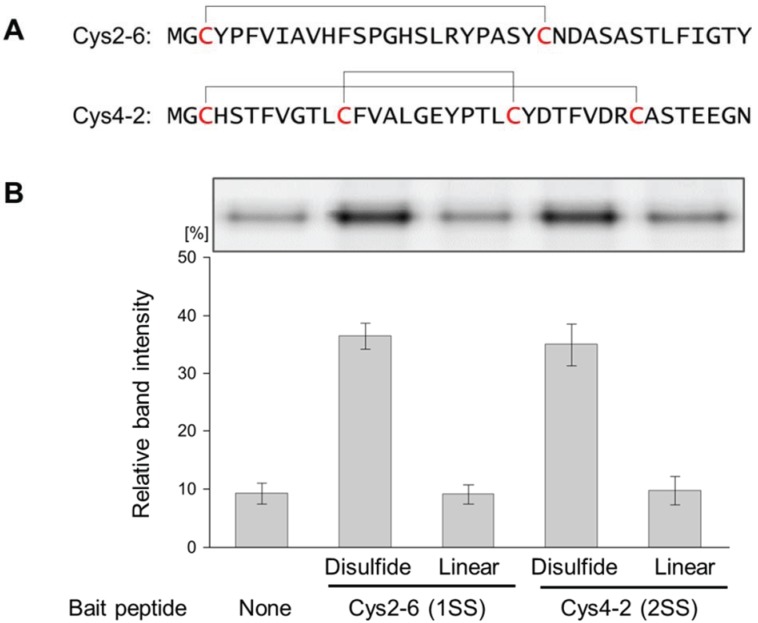
Evaluation of the interaction between the disulfide-containing peptides and sIL-6R by the pull-down method. (**A**) Peptide sequences of Cys2-6 and Cys4-2; (**B**) sIL-6R was pulled down with Cys2-6 (1SS), Cys4-2 (2SS) or the linear forms of each peptide prepared by iodoacetamide treatment (Linear). Magnetic beads with immobilized puromycin-linker only were used as a negative control (None). The pulled-down sIL-6R was visualized by SDS-PAGE (Upper), and the relative band intensities of the pulled-down sIL-6R were measured using analysis software (Lower). Experiments were repeated 3 times. Error bars = standard deviation.

Two disulfide-containing peptide aptamers against sIL-6R: Cys2-6 and Cys4-2, which have one or two disulfide bridges, were used as model disulfide-containing peptides [17]. Each biotin-attached peptide was prepared and immobilized on SA-beads as described in the Experimental section (Figure 1). Then, fluorescein-labeled sIL-6R was pulled down with the SA-beads and detected by SDS-PAGE analysis. Larger quantities of sIL-6R were pulled down by SA-bead-immobilized-Cys2-6 or Cys4-2 peptides compared with the negative control (SA-bead-immobilized puromycin-linker; Figure 3). Furthermore, the iodoacetamide treatment of these disulfide-containing peptides that transformed them into linear peptides decreased the quantities of the pulled-down sIL-6R to the same level as that of the negative control. These results show that the pull-down method can be used to confirm binding properties of not only linear peptides, but also disulfide-containing peptides, which were exposed to oxidative folding using glutathione and PDI. Oxidative folding conditions were optimized by comparing the effect of pH, temperature, or concentration of redox reagents such as GSH⁄GSSG [24]. The pull-down method can also be applied to explore the optimal oxidative folding conditions of disulfide-rich peptides in nature by comparing the amounts of pulled-down prey proteins using the disulfide-containing peptides prepared under several oxidative folding conditions. Additionally, it was confirmed that the disulfide bridges were indispensable for interactions with sIL-6R. To examine the importance of the disulfide bridges in the disulfide-rich peptides for the peptides’ function, binding assays in the presence of dithiothreitol (DTT) are often performed [25]. However, when the targets contain disulfide bridges as well, it cannot be determined which molecules, disulfide-containing peptides or targets cause the loss of interaction in the presence of DTT. In this pull-down method both peptide forms (linear and cyclic) are easily prepared simultaneously, making it suitable for examining the contribution of the disulfide bridges to the function of the disulfide-containing peptides.

### 3.2. Evaluation of the Interaction between the Chemically cross-Linked Peptide and the Target Protein

**Figure 4:**
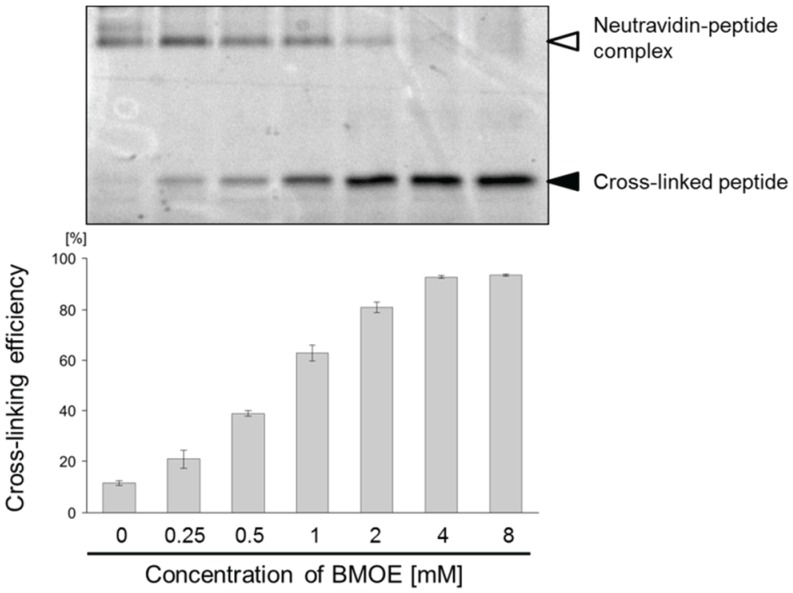
Optimization of the conditions for introducing a chemical cross-linker into the Cys2-6 peptide. Cys2-6 peptide, which was reacted with each concentration of BMOE, was analyzed by SDS-PAGE as described in Figure 2 (Upper). The cross-linking efficiencies of Cys2-6 peptide are indicated as the percentage of the band intensity of the unshifted Cys2-6 peptide against that of the total Cys2-6 peptide (Lower). Experiments were repeated 3 times. Error bars = standard deviation.

**Figure 5:**
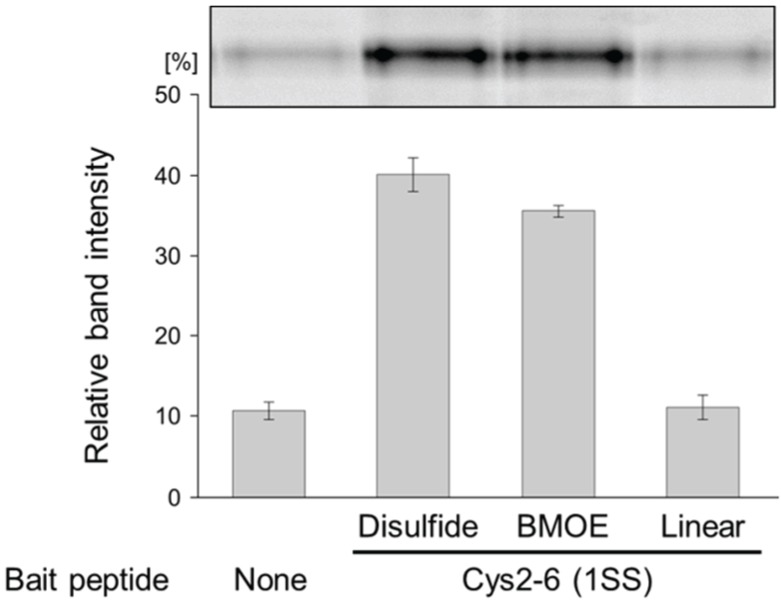
Evaluation of the interaction between the chemically cross-linked Cys2-6 peptide and sIL-6R. Cys2-6 with a disulfide bridge, BMOE, or treated with iodoacetamide (Linear) were prepared, and then sIL-6R was pulled down. The pulled-down sIL-6R was visualized by SDS-PAGE (Upper) and the relative band intensities of pulled-down sIL-6R are shown (Lower). Experiments were repeated 3 times. Error bars = standard deviation.

To expand the application of the pull-down method, we introduced a chemical cross-linker to the bait peptides by a posttranslational reaction. The bait peptides were displayed on the SA-beads via the C-terminus of the peptides and the puromycin-linker that included a PEG spacer, which may be suitable for posttranslational reactions. As a model experiment, we cross-linked the thiol of the cysteins in the Cys2-6 peptide using BMOE, a chemical cross-linking regent, and evaluated its interaction with sIL-6R. First, we examined the conditions for introducing a chemical cross-linker as a disulfide alternative, into the Cys2-6 peptide prepared with the cell-free translation system. The chemical cross-linking efficiency was evaluated easily using a puromycin-linker that contains ribose G to release the bait peptides from the beads according to the scheme in Figure 2B [26]. As the concentration of BMOE increased, the ratio of the Cys2-6 peptide cross-linked with BMOE gradually increased (Figure 4). The cross-linking efficiency reached about 90% under 4 mM BMOE. The interaction of the BMOE-cross-linked Cys2-6 peptide with sIL-6R was confirmed by the pull-down method mentioned above. The quantity of sIL-6R pulled down by the BMOE-cross-linked Cys2-6 peptide was slightly lower than that pulled down by the native Cys2-6 peptide (Figure 5). This result indicates that the affinity of the BMOE-cross-linked Cys2-6 peptide to the protein decreased compared with that of the native peptide. Although the overall conformation of the Cys2-6 peptide could not have changed by the substitution of the disulfide bridge with BMOE, a slight difference in the inter-thiol length can affect the conformation around the binding region of the Cys2-6 peptide with sIL-6R. Alternatively, the Cys2-6 peptides that reacted with two BMOE may have been partially yielded, resulting in decreased quantity of pulled-down sIL-6R.

Introduction of chemical cross-linking into a peptide containing multiple disulfide bonds can be performed by this method, but that could be more complicated because of possibility with several cross-linking patterns. However, an optimal condition for introduction of multiple cross-linkers can be analyzed by combination of chemical cross-linked peptide preparation technique described this work and Time-of-flight mass spectrometry analysis.

Here, we showed that the pull-down method can be applied to assay peptides that require posttranslational modifications, such as oxidative folding and chemical cross-linking reactions. Recently, the presence of a variety of ribosomally synthesized and post-translationally modified peptides has been revealed by the genome sequencing efforts during the first decade of the 21st century [27]. Post-translational modifications of peptides have received the most attention, because they confer attractive properties as drug leads such as better target recognition and high metabolic and chemical stability to peptides [27,28]. The pull-down method may help the study of binding properties, stability and synthesis mechanism of post-translationally modified peptides. Furthermore, cyclic peptides that were cyclized with a non-natural amino acid or chemical cross-linking reagents have been engineered using mRNA display method [29,30]. In this pull-down method the bait peptides can be prepared with several cell-free translation systems with the same procedure as that during the selection cycle, thus it can be used to assay many candidate clones selected from a library easily, rapidly and simultaneously.

## 4. Conclusions

Generally, the preparation of disulfide-rich peptides is complicated, because oxidative folding is required. In this study, we showed that a pull-down method using a puromycin-linker and a cell-free translation system could be easily used to analyze the binding properties of several kinds of disulfide-rich peptides in addition to linear peptides. Using this method, if there are mRNAs coding disulfide-rich peptides, the affinity of these disulfide-rich peptides to the target molecules can be simultaneously confirmed within one day. Furthermore, we demonstrated that the pull-down method could be applied to analyze post-translationally modified peptides. Thus, as a useful molecular interaction analysis method, this pull-down method can promote the discovery of novel beneficial disulfide-rich peptides from natural sources, and the design of functional constrained peptides including disulfide-rich peptides as drug leads or diagnostic regents.
